# Deciphering the recent phylogenetic expansion of the originally deeply rooted *Mycobacterium tuberculosis* lineage 7

**DOI:** 10.1186/s12862-016-0715-z

**Published:** 2016-06-30

**Authors:** Solomon A. Yimer, Amine Namouchi, Ephrem Debebe Zegeye, Carol Holm-Hansen, Gunnstein Norheim, Markos Abebe, Abraham Aseffa, Tone Tønjum

**Affiliations:** Department of Microbiology, Oslo University Hospital, PO Box 4950, NO-0424 Oslo, Nydalen Norway; Uni Research Environment, Centre for Applied Biotechnology, PO Box 7803, N-5020 Bergen, Norway; Infection Control and Environmental Health, Norwegian Institute of Public Health, PO Box 4404, 0403 Oslo, Nydalen Norway; Armauer Hansen Research Institute, Jimma Road, PO Box 1005, Addis Ababa, Ethiopia; Faculty of Medicine, University of Oslo, PO Box 1171, Blindern, 0318 Oslo Norway

**Keywords:** *Mycobacterium tuberculosis*, Lineage 7, Whole genome sequencing, Single nucleotide polymorphism, Mutations, 3R genes, Amhara Region, Ethiopia

## Abstract

**Background:**

A deeply rooted phylogenetic lineage of *Mycobacterium tuberculosis* (*M. tuberculosis*) termed lineage 7 was discovered in Ethiopia. Whole genome sequencing of 30 lineage 7 strains from patients in Ethiopia was performed. Intra-lineage genome variation was defined and unique characteristics identified with a focus on genes involved in DNA repair, recombination and replication (3R genes).

**Results:**

More than 800 mutations specific to *M. tuberculosis* lineage 7 strains were identified. The proportion of non-synonymous single nucleotide polymorphisms (nsSNPs) in 3R genes was higher after the recent expansion of *M. tuberculosis* lineage 7 strain started. The proportion of nsSNPs in genes involved in inorganic ion transport and metabolism was significantly higher before the expansion began. A total of 22346 bp deletions were observed. Lineage 7 strains also exhibited a high number of mutations in genes involved in carbohydrate transport and metabolism, transcription, energy production and conversion.

**Conclusions:**

We have identified unique genomic signatures of the lineage 7 strains. The high frequency of nsSNP in 3R genes after the phylogenetic expansion may have contributed to recent variability and adaptation. The abundance of mutations in genes involved in inorganic ion transport and metabolism before the expansion period may indicate an adaptive response of lineage 7 strains to enable survival, potentially under environmental stress exposure. As lineage 7 strains originally were phylogenetically deeply rooted, this may indicate fundamental adaptive genomic pathways affecting the fitness of *M. tuberculosis* as a species.

**Electronic supplementary material:**

The online version of this article (doi:10.1186/s12862-016-0715-z) contains supplementary material, which is available to authorized users.

## Background

Tuberculosis (TB) has been a major cause of morbidity and mortality among humans for millennia. Each year, approximately 9 million people contract TB and 1.5 million die from the disease [1]. TB is caused by bacterial strains belonging to the *Mycobacterium tuberculosis* complex (MTBC). Whole genome sequencing (WGS) analysis classifies MTBC into seven main lineages (lineages 1–7); lineages 2, 3 and 4 belong to the evolutionary modern group and are considered more recently diversified compared to the ancient lineages of 1, 5 and 6 [2]. An improved understanding of the evolutionary constraints and facilitators on natural populations of MTBC strains is required to develop TB control strategies that efficiently consider the dynamics of mycobacterial evolution.

MTBC and the human host have a long-term co-evolutionary relationship. It is presumed that *M. tuberculosis* originated in Africa and co-evolved into modern lineages with the out-migration of humans from Africa 70–80 thousand years ago [3]. The lineage distribution among cases caused by *M. tuberculosis* exerts distinct geographical associations worldwide [2, 4]. While lineages 1 and 3 are prevalent in East Africa, Central, South- and South-East Asia, lineages 2 and 4 are the most widely distributed worldwide. Lineages 5 and 6, which are also known as *M. africanum* West Africa 1 and West Africa 2, respectively, are localized in West Africa [5, 6]. Lineage 7 is a *M. tuberculosis* lineage recently discovered in north-western Ethiopia and among Ethiopian immigrants in Djibouti [7–10].

We previously investigated the clinical relevance of *M. tuberculosis* lineage 7 as compared to other lineages and found that lineage 7 is associated with prolonged patient delay and slow growth in vitro [11]. Furthermore, phylogenetic characterization of lineage 7 strains by mycobacterial interspersed repetitive unit-variable-number tandem-repeat (MIRU-VNTR) revealed deep phylogenetic branching and recent expansion of this lineage [11]. Factors that may have contributed to the recent expansion are not known. It also remains to be determined which factors may have contributed to growth rate and how this relates to the maintained fitness of lineage 7 strains.

Thirty *M. tuberculosis* lineage 7 strains were subjected to WGS. The genomic profiles were analyzed, evolution was characterized and potential drivers of the recent phylogenetic expansion were identified. Single nucleotide polymorphisms (SNPs) specific to lineage 7 strains were specified. We focused specifically on the presence of repair, recombination and replication (3R) gene mutations relative to the timing of pre- and post-expansion, and on mutations that may be associated with the success of slow-growing lineage 7 *M. tuberculosis* strains.

## Methods

### Bacterial strains, genotyping and drug susceptibility testing

This study included 30 *M. tuberculosis* isolates that were cultivated from sputum samples collected from pulmonary TB patients presenting at selected health care facilities in the Amhara Region of Ethiopia during the period 2008–10 as previously described [9, 10]. The study was approved by the Regional Committee for Medical Research Ethics in Eastern Norway (REK Øst) and the Ethiopian Science and Technology Ministry in Addis Ababa, Ethiopia. Written informed consent was obtained from the study participants before the study was commenced.

Strains defined as lineage 7 were identified by spoligotyping as SIT910 and SIT1724 [9] as previously described [3, 8]. The strains were transferred to Oslo University Hospital, Norway and checked for purity by culturing on Middlebrook 7H10 agar, chocolate agar, and MGIT™ Middlebrook 7H9 in a BACTEC™ 960 (BD, USA) following the manufacturer’s instructions. Drug susceptibility testing (DST) was performed by the proportional absolute concentration [12] and BACTEC™ MGIT™ 960 (BD, USA) following the manufacturer's instructions [13].

### DNA isolation and whole genome sequencing

Genomic deoxyribonucleic acid (gDNA) was isolated from *M. tuberculosis* lineage 7 strains grown on Middlebrook 7H10 agar according to standard procedures [14]. Genomic libraries were paired-end sequenced using the MiSeq Gene and Small Genome Sequencer (Illumina, USA) according to the manufacturer’s specifications (GATC Biotech AG, Germany). Samples were prepared to produce a mean fragment size ~300 bp. To optimize downstream analyses, quality control was performed using the Qualimap [15] and FASTQC programs (http://www.bioinformatics.babraham.ac.uk/projects/fastqc/). Sequence data have been deposited in the European Nucleotide Archive with the study accession code PRJEB13960.

### Bioinformatics analyses

Paired-end lineage 7 genome sequence reads were mapped to the genome sequence of the *M. tuberculosis* H37Rv reference strain (version NC_000962.3) using BWA aligner [16]. The genome sequence mapping results were visualized according to the WGS of the H37Rv reference strain and its genome annotation using Unipro UGENE. In order to identify SNPs uniquely associated with lineage 7 strains, the sequences were compared with those available from previously sequenced MTBC strains stored on publicly available databases (Additional file 1). Comparative SNP typing was performed on 161 isolates (33 lineage 7 and 128 representative of lineages 1–6) using the Unified Genotyper of the Genome Analysis Toolkit (GATK). In-house Python modules were applied to all generated Variant Call Format (VCF) files in parallel to comparatively analyse and filter the SNPs detected, and to produce a comparative multiple sequence alignment of all positions for which a SNP was called in at least one of the strains in the complete dataset. SNPs were retained if they were supported by 5 reads with a quality control (QC) score ≥ 30. SNPs in PE/PGRS genes, mobile elements, and those linked to insertion/deletion regions were excluded from the analysis. Indels were mapped by combining BreakDancer [17] and Pindel [18] outputs. All DNA sequence structural variations identified were inspected manually. Genes harbouring nsSNPs or indels were grouped according to the different classes of the Clusters of Orthologous Groups (COG) classification [19, 20]. Deletions were visualised using the matplotlib library.

### Phylogeny and evolutionary predictions

Phylogeny was inferred using RaxML (version 8.1.3). RaxML was used for Maximum likelihood (ML) based estimates of the MTBC phylogeny and 1000 bootstrap replicates were performed to assess statistical support. The phylogenetic trees were visualized using FigTree (version 1.4.0). The substitutions leading to each SNP were mapped to the phylogenetic tree using Mesquite version 3.02 using the parsimony ancestral state reconstruction method (Mesquite: a modular system for evolutionary analysis, Version 3.02) [21]. Identification of the ML-based common ancestor (MLCA) and the presence of a clock-like signal in this dataset were investigated by plotting the root-to-tip distance against time using a linear regression model using Path-O-Gen software.

### Statistical analysis

The binomial test was used to compare the sSNPs vs nsSNPs distribution of COG categories in lineage 7 strains. We took into account the number of SNPs in each category and the total length of the genes where mutations are located. For each cell, the binomial test was calculated using Excel as follows: BINOMIAL DIST (Number of SNPs, Total number of SNPs, gene length/Total gene length, 1).

## Results

### MTBC lineage 7 strains are originally deeply rooted in the phylogenetic tree

**Fig. 1:**
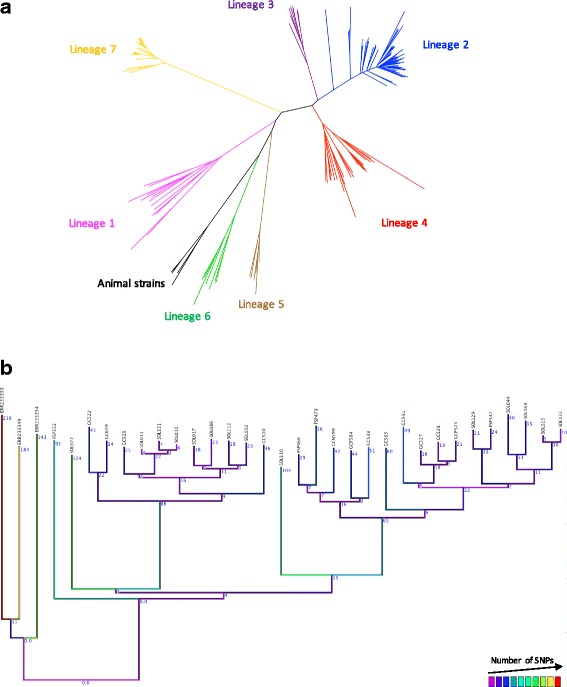
**a** Maximum likelihood (ML) tree including strains belonging to all MTBC lineages. The ML tree is based on all identified polymorphic sites. **b** ML tree of lineage 7 isolates included in this study

A phylogenetic tree was built based on the complete number of SNPs extracted from genomic DNA sequences as compared to a diverse set of whole genome sequences from 161 MTBC WGS (Fig. 1a). Lineage 7 strains form a distinct group which is positioned deeply between the “ancient” and evolutionary “modern” lineages. Furthermore, lineage 7 strains were shown to exhibit a recent expansion (Fig. 1a). Bayesian statistical methods employed to estimate the time of the primary lineage 7 expansion suggested that it started approximately 310 years ago (Additional file 2).

### Lineage 7 strains exhibit a high number of nsSNPs in metabolic genes

A total of 3215 SNPs were observed in lineage 7 when compared to other MTBC lineages (Fig. 1b). The proportions of synonymous SNPs (sSNPs) and non-synonymous SNPs (nsSNPs) distribution in the lineage 7 isolates were compared. sSNPs and nsSNPs accounted for 1140 (35.45 %) and 2075 (64.5 %) events, respectively, with an overall SNP ratio of 1.8. The number of SNPs in intergenic regions was 334.

**Fig. 2:**
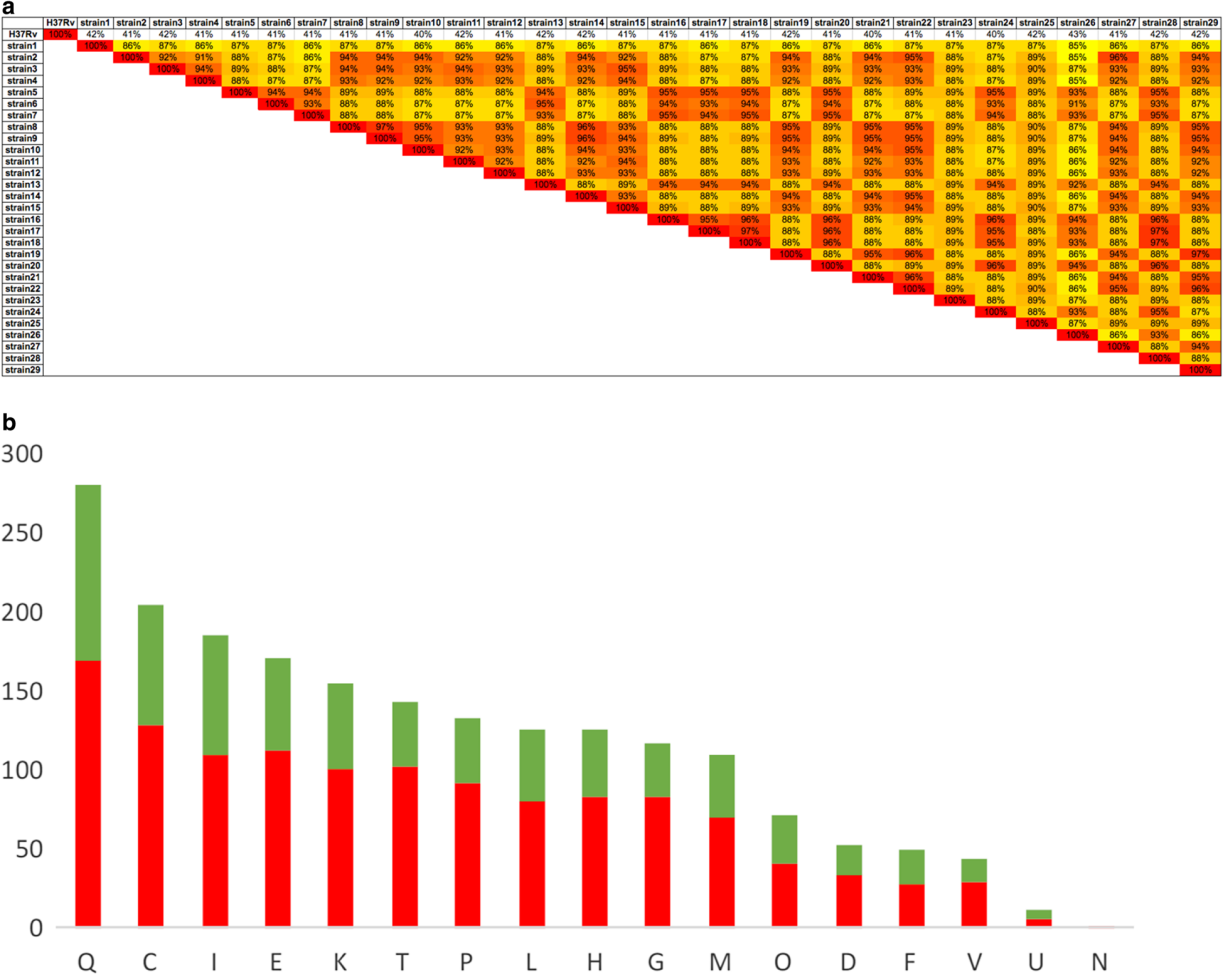
**a** Distribution of all identified SNPs in lineage 7 strains based on the Cluster of Orthologous Classification (COG). The green color defines the synonymous SNPs and the red color the non-synonymous SNPs. **b** Similarity matrix based on whole genome analysis. The percentages indicated in each box correspond to the identity between two isolates at the genomic level. Letter codings are described as follows: [C] Energy production and conversion, [D] Cell cycle control, cell division, chromosome partitioning, [E] Amino acid transport and metabolism, [F] Nucleotide transport and metabolism, [G] Carbohydrate transport and metabolism, [H] Coenzyme transport and metabolism, [I] Lipid transport and metabolism, [K] Transcription, [L] Replication, recombination and repair, [M] Cell wall/membrane/envelope biogenesis, [N] Cell motility, [O] Post-translational modification, protein turnover, and chaperones, [Q] Secondary metabolites biosynthesis, transport, and catabolism, [T] Signal transduction mechanisms, [U] Intracellular trafficking, secretion, and vesicular transport, [V] Defense mechanisms

More than 800 SNPs specific to lineage 7 isolates were identified when compared to the WGS of isolates that belong to the different MTBC lineages. The SNP distribution in the lineage 7 strains showed variation as shown in Fig. 2a.

We analyzed the distribution of the total 3215 SNPS observed in lineage 7. Accordingly, 1974 SNPs were located in the genomic coding regions that were classified in the following cluster of orthologous gene (COG) categories: secondary metabolite biosynthesis, transport, and catabolism (Q) (*n* = 280); energy production and conversion (C) (*n* = 205); lipid transport and metabolism (I) (*n* = 185); amino acid transport and metabolism (E) (*n* = 170); transcription (K) (*n* = 154); signal transduction mechanisms (T) (*n* = 143); inorganic ion transport and metabolism (P) (*n* = 132); replication, recombination, and repair (L) (n 126); coenzyme transport and metabolism (H) (*n* =125); carbohydrate transport and metabolism (G) (*n* = 116); cell wall/membrane/envelope biogenesis (M) (*n* = 109); posttranslational modification, protein turnover, and chaperones (O) (*n* = 72); cell cycle control, cell division, chromosome partitioning (D) (*n* = 52); nucleotide transport and metabolism (F) (*n* = 50); defense mechanisms (V) (*n* = 43); intracellular trafficking, secretion, and vesicular transport (U) (*n* =12) (Fig. 2b and Table 1).

**Table 1:** Distribution of SNPs according to the Clusters of Orthologous Groups (COG) classification. The binomial test was calculated using Excel as follows: BINOMIAL DIST (Number of SNPs, Total number of SNPs, Gene length/Total gene length, 1)

Cluster of Orthologous Categories	COG codes	Nb of nsSNPs	Nb of sSNPs	Total nb of SNP	Nb of genes	Total gene length	BINOMIAL TEST
Secondary metabolites biosynthesis, transport and catabolism	Q	169	111	280	85	186303	0.016339104
Energy production and conversion	C	128	77	205	104	141315	0.00956263
Lipid transport and metabolism	I	110	75	185	86	108423	0.551376551
Amino acid transport and metabolism	E	112	58	170	85	111717	0.075317667
Transcription	K	100	54	154	68	60555	0.999999395
Signal transduction mechanisms	T	102	41	143	43	55596	0.99999919
Inorganic ion transport and metabolism	P	92	40	132	70	100593	0.000946095
Replication, recombination and repair	L	80	46	126	51	75246	0.463792377
Coenzyme transport and metabolism	H	83	42	125	60	63582	0.955338675
Carbohydrate transport and metabolism	G	83	33	116	57	82752	0.017486939
Cell wall/membrane/envelope biogenesis	M	70	39	109	57	70737	0.164200506
Post-translational modification, protein turnover, and chaperones	O	41	31	72	42	54294	0.016391663
Cell cycle control, cell division, chromosome partitioning	D	34	18	52	22	38130	0.059214452
Nucleotide transport and metabolism	F	28	22	50	33	36285	0.073929704
Defense mechanisms	V	29	14	43	26	34749	0.017540819
Intracellular trafficking, secretion, and vesicular transport	U	6	6	12	7	8544	0.310996283
Cell motility	N	1	0	1	0	0	1
Not in COGs	R	441	228	669	386	325323	0.999999959
General function prediction only	R	250	136	386	189	238986	0.156980544
Function unknown	S	116	69	185	106	101895	0.838428538

Lineage 7 strains exhibited a high proportion of mutations inducing an amino acid change in genes involved in carbohydrate transport and metabolism, energy production and conversion, defense mechanisms, secondary metabolites biosynthesis, transport and catabolism, inorganic ion transport and metabolism, and post-translational modification, protein turnover, and chaperone. In contrast, a low frequency of mutation was observed in genes involved in nucleotide transport and metabolism, intracellular trafficking, secretion, and vesicular transport, and cell motility (Fig. 2b and Table 1).

**Fig. 3:**
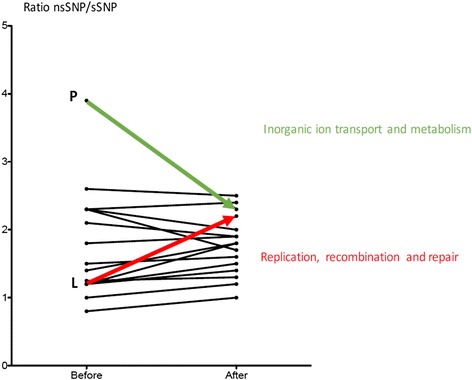
Comparison of the ratio of nsSNP/sSNP. Observed differences in the ratio of nsSNP/sSNP before and after the expansion of lineage 7

To elucidate drivers of recent lineage 7 expansion, mutations in major COG categories before and after the phylogenetic expansion period were compared. Accordingly, the number of nsSNPs in 3R genes (COG category L) were significantly higher after than before the expansion started (nsSNP/sSNP ratios of 2.3 and 1.2, respectively, P-value < 0.05, *χ*^2^ test) (Fig. 3).

The number of nsSNPs in genes involved in inorganic ion transport and metabolism were significantly higher before than after the expansion began (nsSNP/sSNP ratios of 3.9 and 2.5, respectively, P-value < 0.05, *χ*^2^ test) (Fig. 3). Among the genes involved in inorganic ion transport and metabolism (COG category P), *polyphosphate kinase* (*PPK1*) exhibited a high number of nsSNP (Additional file 3). A high number of nsSNPs were also observed in the following genes: *dnaB and Rv2090* (3R genes), *glpk* (gene involved in glycerol metabolism); *pks12* and *pks8* (genes involved in secondary metabolites biosynthesis, transport and catabolism); *mmpl12*, *mmpl4* and *mmpl3* (genes involved in fatty acid transport); and *mbtE* and *accA3* (genes involved in long fatty acid synthesis) (Additional file 3).

### Deletion analyses

**Fig. 4:**
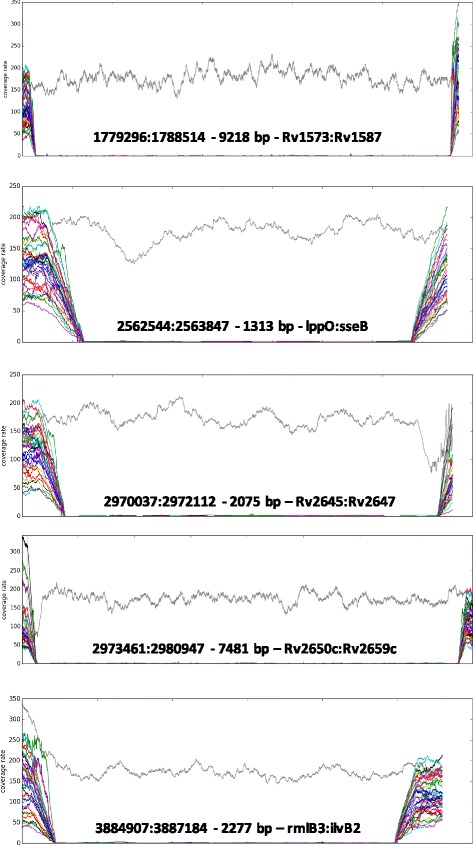
List of specific deletions identified in all lineage 7 isolates included in this study. Deletions were identified by calculating the coverage rate throughout the genome using bedtools on the alignment files generated by samtools. Each line corresponds to the coverage rate for each strain. The gray line corresponds to a control strain that does not include any of the identified deletions specific for the lineage isolates. For each deletion, the information is: genomic coordinates, size of the deleted region and the genes concerned.

A total of 22346 bp deletion events occurred along the WGS phylogeny. The specific sites of the deleted genes including their functional categories were: *Rv2650c-Rv2659c* (insertion sequences and phages)*; lppO or Rv2290* (cell wall and cell processes); *sseB or* Rv2291 (intermediary metabolism and respiration)*; rmlB3 (Rv 3468), mhpE (Rv3669), ilvB2 (Rv 3470) (*intermediary metabolism and respiration)*; Rv2645-Rv2647* (insertion sequences and phages*); Rv2645 (*unknown functional category)*; Rv2646* (insertion sequences and phages); and *Rv1573-Rv1587(*insertion sequences and phages) (Fig. 4).

## Discussion

This is the first genomic study that provides an insight into the recent evolution and drivers of fitness for survival among *M. tuberculosis* lineage 7 strains. Genomic phylogenetic tree analysis positioned lineage 7 between “ancient” and “modern” lineages, confirming the findings in previous MIRU-VNTR studies [5, 6]. More than 800 SNPs specific to *M. tuberculosis* lineage 7 strains were identified, indicating that the bacterium was accumulating specific mutations for a long time before the phylogenetic expansion began.

In contrast to the deeply rooted *M. canettii*, which grows faster than MTBC strains of other lineages [22], lineage 7 strains grow slowly in vitro [11]. To identify SNPs that potentially could be associated with the expansion event, the proportion of nsSNP and sSNP mutations in functional components according to COG were compared. Mutations in genes involved in inorganic ion transport and metabolism were significantly higher before rather than after the expansion started while mutations in 3R genes were significantly higher after the expansion began. The significant increase of nsSNPs in 3R genes corroborates our previous finding on *M. tuberculosis* adaptive responses [23–25] and may be related to fitness for survival. This could be a consequence of selected critical mutations induced at a specific time point, presumably at an early stage of the lineage 7 expansion, leading to a transient or constitutive adapted mutator phenotype.

Other factors may also have contributed to the recent expansion. Hosts with reduced immune competence due to poor nutrition could have facilitated lineage 7 strains to evolve rapidly leading to more mutations and hence diversity. In addition, poor living conditions, frequent drought and rapid population growth in the country may have given lineage 7 ample opportunity for diversification.

The significant increase in nsSNP in inorganic ion transport and metabolism before the expansion period may indicate a coping strategy adopted by lineage 7 strains against a potential environmental stress factor to which the bacteria were exposed. *M. tuberculosis* may encounter a multitude of stress factors (e.g. oxidative, acidic, nutrient, membrane damage, heat shock and ribosomal stress factors) when interacting with the host that potentially induce adaptive responses enabling improved survival [26]. Specifically, the high proportion of nsSNP observed in the *polyphosphate kinase 1* (*PPK1*) gene may indicate possible exposure of lineage 7 strains to stressful environmental conditions. Previous studies showed that the *PPK1* gene plays a crucial role in bacterial survival under conditions of stress including lag-phase, under nutrient starvation and oxidative stress [27, 28]. It may be speculated that lineage 7 strains accumulated mutations to adapt to such types of stress before the recent expansion started. An earlier study showed that bacteria without the ability to adapt to oxidative and nitrosative stress grow slowly in macrophages and are likely to die [29]. Additional transcriptomic and proteomic studies are warranted to further investigate the adaptive responses of lineage 7 strains to stress factors that are likely to be associated with the timing of the recent expansion. A significant decrease of nsSNPs in genes involved in inorganic ion transport and metabolism was observed after the recent expansion of lineage 7 started (Fig. 3). Very low levels of nsSNPs have previously been attributed to the effect of purifying selection [30].

A high frequency of nsSNPs mutation in the *dnaB* gene was observed. A former biochemical study indicated that the *dnaB* gene plays an important role in both initiation and elongation of DNA helicase [31]. A very high proportion of nsSNPs in the *glpk* gene, involved in glycerol utilization [6], was also observed. An earlier study identified the *glpk* gene as one of the 42 growth-attenuating genes in *M. tuberculosis* [32]. Therefore, the high frequency of mutations observed in this gene may be linked to the slow in vitro growth of lineage 7 strains reported in our previous study [11]. Further investigations into the enzymatic function of these genes are warranted to analyze the effect on in vitro growth of lineage 7 stains.

A number of gene deletions specific to lineage 7 strains were observed. One area of deletion was in the regions of *Rv3468-Rv3470.* Valine and isoleucine biosynthesis that involve *Rv3470* [32] are essential pathways required for optimal growth of the bacteria. The deletion of *RV3470* is thus likely to have contributed to the phenotypic consequences of slow in vitro growth among lineage 7 strains [11].

To date, the distribution of the “ancient” *M. tuberculosis* lineages, 5, 6 and *M. canettii* is restricted to Africa, now supplemented by lineage 7. It is not known why these three lineages are found only in specific regions of Africa. The ability to cause secondary cases and/or outbreaks is considered to be a measure of fitness or success in MTBC transmission. Given the restricted geographic distribution, “ancient” *M. tuberculosis* lineages are not as successful as “modern” lineages. Despite the fast-growing nature of *M. canettii* as compared to other strains, only 60 *M. canettii* strains have been recognized to date [33]. Ancient lineages are being replaced by modern strains; the prevalence of *M. africanum* West African 2 in Guinea-Bissau decreased from 51 % to 39 % between 1989 and 2008 [34]. The prevalence of *M. africanum* lineages in other countries including Côte d’Ivoire, Ghana and Cameroon is also declining [35–38]. More studies are needed to characterize the transmission pattern of the recently identified *M. tuberculosis* lineage 7 strains.

Results of a previous study indicated that natural variation among clinical isolates may change epidemiologic patterns in a population [39]. Infection with *M. tuberculosis* strains that illicit pro-inflammatory cytokines are very well controlled in healthy persons with effective innate responses [39]. In contrast, another study demonstrated that a slower growing *M. tuberculosis* strain causing a less protective innate immune response may more effectively elicit active disease and increase transmission in the community [40]. Lineage 7 accounted for 16 % of the distribution and was the second largest cluster among the strains collected in our previous study [9]. This shows that lineage 7, despite growing slowly in vitro, is responsible for significant transmission in a heterogeneous community. This may indicate that the fitness of lineage 7 strains results in relatively efficient transmission of TB.

Lineage 7 is prevalent in the Amhara Region of Ethiopia (9). An earlier study by Firdessa et al. [8] reported lineage 7 strains from the Woldiya area of the Amhara Region. Genotyping data from the national prevalence survey in Ethiopia reported two SIT910 lineage 7 strains from East Gojjam Zone of the Amhara Region [41]. Our study included a higher number of lineage 7 strains than any other study to date, which might suggest that the Amhara Region of Ethiopia may be the cradle of *M. tuberculosis* lineage 7.

## Conclusions

TB caused by *M. tuberculosis* lineage 7 strains is an emerging disease in Ethiopia and the Horn of Africa. Due to the high mobility and migration of people in this region, the presence of ecological and individual risk factors, and the increasing trend of surveillance, it is likely that the number of MTBC lineage 7 cases diagnosed will increase.

This study identified unique genomic signatures associated with MTBC lineage 7 strains and identified SNPs in genes possibly related to the clinical and microbiological features observed. We suggest that the relatively high proportion of nsSNPs in 3R genes may have contributed to the recent phylogenetic expansion of lineage 7 strains that started approximately 310 years ago. The high frequency of mutations in genes involved in inorganic ion transport and metabolism before the expansion period may indicates an adaptive response of lineage 7 strains to stress factors experienced by the bacteria. The high proportion of nsSNPs and deletions observed in specific genes may have contributed to phenotypic consequences including slow growth. Further functional biochemical studies addressing specific SNPs and gene deletions associated with lineage 7 strains are warranted to delineate the relative association to virulence and relation to clinical presentation.

## Abbreviations

3R genes: DNA repair, recombination and replication genes; COG; clusters of orthologous groups; ML, maximum likelihood; MIRU-VNTR, mycobacterial interspersed repetitive unit-variable-number tandem-repeat; MTBC, mycobacterium tuberculosis complex; nsSNPs, non-synonymous single nucleotide polymorphisms; SIT, spoligo international type; WGS, whole genome sequencing.

## Additional files

Additional file 1: Is a table listing databases used for comparative analysis of WGS. (XLSX 12 kb) Additional file 2: Shows time of the primary lineage 7 expansion. (PDF 8 kb) Additional file 3: Is a table listing the SNPs in genes. (XLSX 11 kb)
