# Supplementary figures and images for: Deciphering the recent phylogenetic expansion of the originally deeply rooted Mycobacterium tuberculosis lineage 7
Source: BMC Evol Biol. 2016 Jun 30;16:146. doi: 10.1186/s12862-016-0715-z (PMC4929747; doi:10.1186/s12862-016-0715-z)

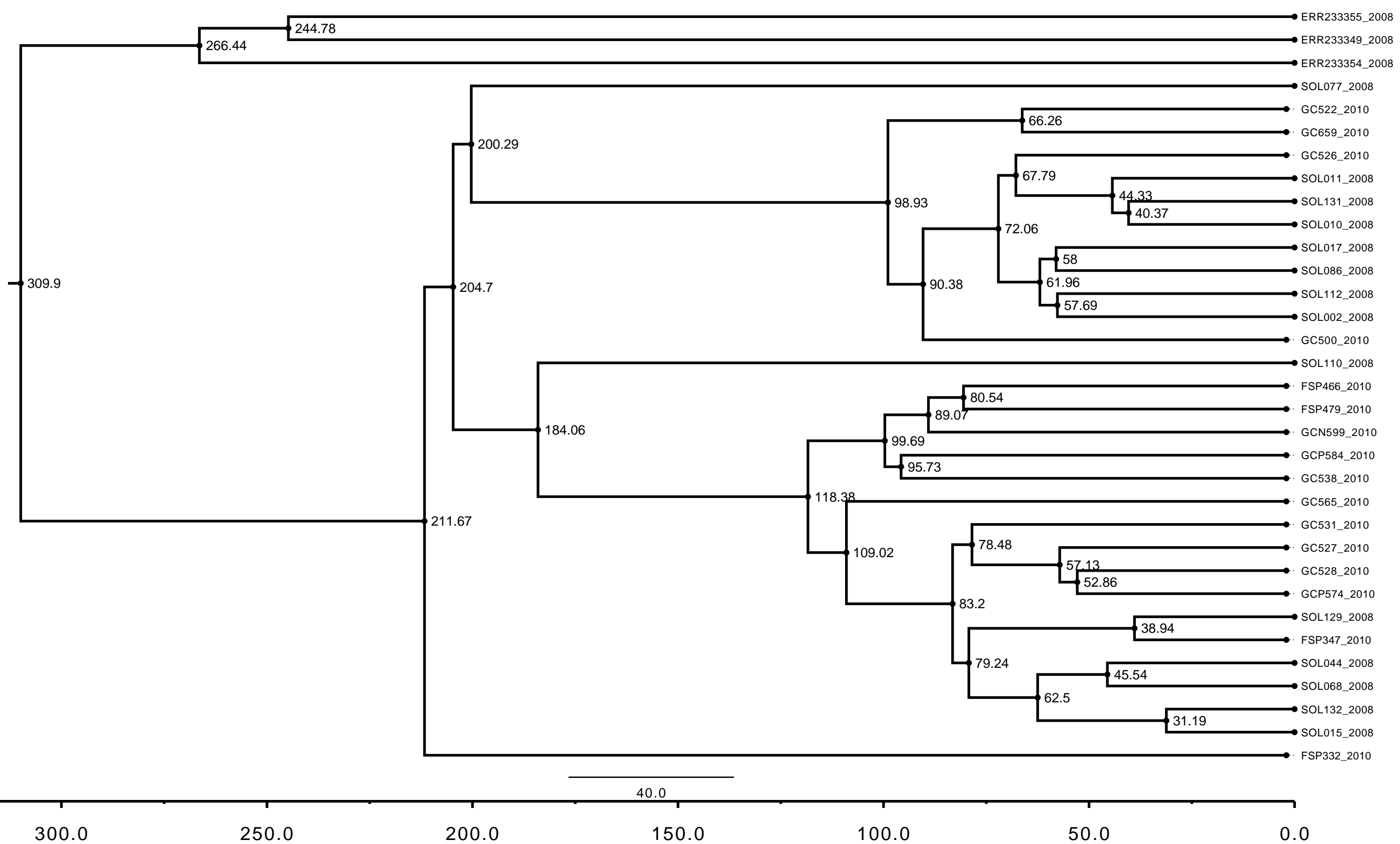

Supplement: Additional file 2: — Shows time of the primary lineage 7 expansion. (PDF 8 kb) [file 12862_2016_715_MOESM2_ESM.pdf]
